# P-CRS: A Clinical Scale to Assess the Parent-Child Relationship in Infancy and Early Childhood

**DOI:** 10.3390/ijerph17103458

**Published:** 2020-05-15

**Authors:** Anna Maria Speranza, Maria Quintigliano, Marco Lauriola, Alexandro Fortunato

**Affiliations:** 1 Department of Dynamic and Clinical Psychology, “Sapienza” University of Rome, 00185 Rome, Italy; maria.quintigliano@uniroma1.it (M.Q.); alexandro.fortunato@uniroma1.it (A.F.); 2 Department of Social and Developmental Psychology, “Sapienza” University of Rome, 00185 Rome, Italy; marco.lauriola@uniroma1.it

**Keywords:** caregiving, infancy, early childhood, parent-child relationship, assessment

## Abstract

This study aimed to examine the ability of a new clinician-report tool, the Parent-Child Relationship Scale (P-CRS), to assess the individual contributions that parents and their children make within the parent-child relationship, as well as interactions between parents and children in terms of developmental psychopathology. As clinical diagnoses in early childhood is both important and difficult, it is necessary to identify tools that can effectively contribute to evaluating parent-child relationships during the diagnostic process. A sample of 268 mother-child dyads, taken from both public and private clinical settings, was assessed. Clinicians were asked to assess these dyads using the P-CRS after four to five sessions of clinical evaluation. The results indicated that the three areas assessed by the P-CRS—“Interaction”, “Child” and “Parent”—could have different impacts on the various aspects of the parent-child relationship within distinct diagnostic groups. Thus, our findings support the use of the P-CRS to assist with clinical diagnosis during early childhood.

## 1. Introduction

From a clinical perspective, making a diagnosis during infancy and early childhood is both important and difficult. Certainly, a significant number of infants and young children exhibit symptoms of psychopathological conditions that often persist, and that need to be identified and addressed rapidly [1]. However, at this age, it is not easy to distinguish between transitory problems and actual disorders [2]. Moreover, in infancy and early childhood, the infant/child-caregiver relationship has a central role and needs to be included in the diagnostic process. Regardless of child symptomatology, relationships could be considered a risk factor or a resource for child socio-emotional development, and therefore, assessment should include the level of global functioning of the relationship; the adaptive flexibility of both the child and the parent; and the conflict levels and capacity for resolution between child and parent, as well as the effect that the relationship can have on development [3].

The Diagnostic Classification Zero to Three-Revised (DC: 0-3R) [4] and its latest version (DC: 0-5) [5], and also the Infancy and Early Childhood (IEC 0-3) Section of PDM-2 [3] have all focused on the importance of multi-informant assessment approaches, particularly during infancy and early childhood. This multi-informant approach has proven to be useful in the assessment of early psychopathology in many studies [6,7,8,9,10,11]. Thus, a clinician-structured point-of-view is seen as a rich source of information for a complete assessment. 

Traditionally, authors [12,13] have widely pointed out that the intersubjective dimension of the parent-child relationship includes something more than individual contributions. Caregiver characteristics, such as attachment state of mind, specific parenting style, and mental health or at-risk behaviors [14] deeply influence parent-child relationships and, consequently, child development [15,16,17]. These authors also underlined that this relationship is determined by a child’s specific characteristics, such as temperament [18], methods of communication and interaction with caregivers or other people, and emotional regulation functioning [19]. All these characteristics are co-built and more or less flexibly adapted into specific parent-child relationships [20]. Furthermore, the parent-child relationship has to be considered not only as a casual dimension that could explain psychopathology, but also as an important factor to understand flexibility, regulation, contingency, and many other aspects of child development, and not least as a basis on which we can assess parent and child resources. 

However, another important research finding should not be overlooked: mother-child and father-child interactions could be very different [21], and there are several cases in which a child’s pathological behaviors only appear in a specific caregiving relationship and cannot be generalized to others. Thus, it could be important to consider this aspect when conducting an assessment [5], then carefully evaluate each relationship. 

The parent-child relationship is usually assessed using three main methods: self-reports, interviews, and observational scales. Self-reports are used to measure difficulties with the parenting role (Parenting Stress Index Short Form) [22], specific educational approaches used with the child (Parenting Styles and Dimensions Questionnaire) [23,24,25], or feelings about parenting roles and their own children [26]. Interviews are focused on a representational level and can be focused on a parent’s probable experience with his or her own parents, such as the Adult Attachment Interview [27], or on parental representations of the child, of themselves as parents, and of the parent-child relationship, such as the Parent Development Interview [28,29]. Lastly, observational methods are used to assess interactions between parents and children over several sessions [30,31,32], and in more or less structured sessions [33,34]. 

Thus, given the importance of a multi-informant assessment at this age, and considering clinician evaluation as an important support to this assessment, our aim was to create an instrument that is able to identify key aspects useful for a complete evaluation, by focusing on the parent-child relationship. Some authors have underlined how research has mainly focused on school-age children instead of infancy and early childhood, even if there is a high prevalence of behavioral and emotional problems during the earlier developmental stage [35,36]. 

Therefore, the aim of this study is to, first, develop a new clinician-report tool, to assess the parent-child relationship during infancy and early childhood. Three particular areas will be investigated through the Parent-Child Relationship Scale (P-CRS): the parent, the child, and the parent-child interaction. Second, this study aims to validate the P-CRS in a clinical sample assessed by expert clinicians, evaluating the factor structure of each area of the P-CRS, identifying dimensions that are useful to describe the parent-child relationship with specificity. Finally, the last aim of this study is to test the hypothesis that these dimensions could be compared among different psychopathological groups, and could capture peculiar characteristics of the parent-child relationship within that specific pathological framework, consistent with the scientific literature on this topic. 

The validation of a clinician-report instrument will be relevant for clinicians who have to carry out evaluations of parent-child relationships without using complex tools or standardized procedures.

## 2. Materials and Methods 

### 2.1. Participants and Procedures

This study was conducted among 184 clinicians who had observed 268 mother-child dyads, with one of the following clinical conditions: neurological disease/organic illness/genetics condition (N = 36); autism spectrum disorder (N = 45); developmental delay (N = 96); prematurity (N = 40); affective and relational disorders (N = 23); and other clinical conditions (e.g., feeding disorder, witness of domestic violence, sleeping disorder; N = 28). The last category comprises different residual clinical conditions that have been included in the “other” category, due to the small size of these clinical groups. For this reason, even if they are included in the study, we are not going to consider this last group in the final discussion. Note that inclusion criteria for each group excluded overlapping characteristics between the two groups (e.g., children with delay being born preterm). The dyads observed were composed of 268 mothers (age: M = 35.49 years; SD = 5.77 years) and 175 boys and 93 girls, ranging in age from 5 months to 6 years (M = 43.52 months; SD = 18.66 months). Mothers’ mean ages distribution was not significantly different between the six clinical conditions. Most of the mothers were Italian (81.3%) and married, or living together with a partner (86.9%); 36.2% were employed; 40.3% were homemakers; 10.8% were self-employed; and 5.6% were unemployed. The other 7.1% did not answer the employment question. With respect to the educational level, 1.5% went to elementary school, 14.2% to middle school, 53.0 to high school, and 31.3% had a university degree. Children’s mean ages distribution was different between the six clinical groups: in particular, all the groups have the same mean excepting for “prematurity” condition, in which children were significantly younger than in all the other groups (M = 16.03 months; SD = 10.33 months) and for “neurological disease/organic illness/genetics condition” (M = 42.19 months; SD = 18.48 months), in which children were significantly younger than in “developmental delay” (M = 51.94 months; SD = 15.45 months).

Participants were informed by the clinicians who rated the scale about guaranteeing anonymity and confidentiality. Clinicians also collected written informed consent from all mothers in this study. The current study has received approval from the Ethical Committee of the Department of Dynamic and Clinical Psychology (Faculty of Medicine and Psychology, Sapienza University of Rome). All procedures performed in studies involving human participants were in accordance with the ethical standards of the institutional and/or national research committee and with the 1964 Helsinki declaration and its later amendments or comparable ethical standards. Informed consent was obtained from all individual participants included in the study.

The clinicians (118 women and 66 men), each with at least 10 years of clinical experience, were contacted through local services (45.1%), hospitals (18.5%), private centers (17.4%), residential facilities (9.2%), forensic centers (6.0%), and other clinical centers (3.8%). They varied in theoretical approaches: 36.4% psychodynamic, 18.5% cognitive behavioral, 22.3% systemic, 12.5% integrated, and 10.3% other approaches. They were asked to give responses for P-CRS items, referring to each child diagnosed over the course of a year. Clinicians were informed about the importance of their cooperation and were trained by a member of this study’s research team, who also later acted as their supervisor during the study. Observational sessions were settled based on the clinicians’ visits scheduling, usually once a week. It was not required a specific setting because observational sessions were parts of the psychodiagnostic assessment. We usually suggested play or free interactions, lasting about one hour. Note that the P-CRS is not designed strictly as an observational measure, but also as a scale useful to keep in clinician’s mind during the observations and at the end of the assessment for drafting the final report. Therefore, the use of P-CRS disregards the specific procedure adopted and the clinician’s theoretical orientation. Observations were carried out by clinicians in vivo.

### 2.2. Development and Features of the P-CRS

The early mother-child relationship and its influence on current or later development are not easy phenomena to investigate. The P-CRS aims to assess the quality of this relationship, particularly during infancy and early childhood, then associate specific relational patterns with possible psychopathological outcomes. The P-CRS is suitable for children from 1-month to 6-years-old. Items have been formulated to be adapted to the entire age range considered.

An important contribution to this measure was offered by Axis II of the DC: 0-3R [4], specifically through the Parent-Infant Relationship Global Assessment Scale (PIR-GAS) and Relational Problem Checklist (RPCL). Some parts of these scales were operationalized into P-CRS items, to provide clinicians with an easy-to-use instrument with low levels of inference.

In order to allow the clinician to evaluate specific aspects of the parent-child relationship, the P-CRS offers three macro-areas based on the specific focus of their items: the child, the parent, and the interaction between parent and child.

After observing a dyad’s interaction in at least four or five sessions, the clinician scores 50 items on a five-point Likert scale, ranging from 1 = not at all descriptive to 5 = strongly descriptive. As previously explained, items are divided into three different areas. “Interaction” includes items focused on the overall parent-child relationship, for example: “The interactions are pleasant for the child and for the parent and without reasons of anxiety” (item 1); “The relationship, even in the absence of conflict, may be inappropriate from the point-of-view of the child’s development” (item 11); and “The parent and the child present an anxious mood observable through motor tension, apprehension, agitation, facial expression, vocalization, or language” (item 32). “Parent” includes items that focus on the parent’s contributions to the relationship through descriptions of abilities or specific behaviors, for example: “The parent is able to fully support the functional capabilities appropriate to the age of the child” (item 5) and “The parent physically manipulates the child in a clumsy way” (item 33). “Child” includes items focused on the child’s contributions to the relationship, for example: “The child has a disability that alters the parent’s ability to maintain an adequate relationship” (item 13) and “The child manifests provocative and aggressive behaviors toward the parent” (item 42). The clinician must answer the questions considering each dyad separately (mother-child or father-child) or one of the two, if he or she has not had the opportunity to observe both.

The items are formulated in order to investigate a wide range of feelings and behaviors, and to determine the functioning and relational dynamics between the child and each of the two parental figures. Items are easy to understand and avoid jargon or technical terms, in order to be answered by clinicians from different trainings and theoretical orientations. Moreover, the items were created with the aim of detecting levels of anxiety, conflict, adaptivity, and problem-solving ability for each member of the dyad, while also delineating psychological involvement, quality of behavior, and affective tone expressed in verbal and non-verbal communication.

### 2.3. Statistical Analyses 

Exploratory factor analysis assumes a multivariate normal distribution. Since examination of the item-response pattern revealed an asymmetric response distribution in most cases (Supplementary Table S1), skewness and kurtosis indices were examined, to detect more severe violations of normality. An established rule-of-thumb is to remove items from factor analysis with univariate skewness and kurtosis above 3 and 10, respectively [37]. Based on these criteria, 13 items were removed (Supplementary Table S1). Note that, for items 1–5, the numerical scoring scale is in the opposite direction from the others, so these items were reverse-scored to calculate factors.

The Kaiser–Meyer–Olkin (KMO) measure of sampling adequacy reflects the proportion of variance among variables that might have common variance and the suitability of a correlation matrix for factor analysis. The KMO attained high values for the Interaction and Parent item sets (0.92 and 0.91, respectively). These values indicated that the common variance in the matrix was adequate. For the Child set, the KMO was mediocre (.66), but still indicated that the sampling was adequate for factor analysis. A Principal Axis (PA) factor analysis with Oblimin rotation was conducted by FACTOR 10.9 on the three separate sets of items (Interaction, Parent, and Child).

## 3. Results

### 3.1. Factor Analysis

There was a total of 22 Interaction items. Four were excluded from factor analysis because they did not show normal distribution. To choose how many factors should be considered, different indices were analyzed. While the eigenvalue-greater-than-one indicated four factors, the scree-plot showed two drops in eigenvalue size after the first and the fourth eigenvalue, and finally, the PA criterion suggested one factor to be retained. This information was interpreted as suggestive of four oblique factors, accounting for 69.43% of the variance in ratings, which were subsequently interpreted. Dysfunctional Relationship (Cronbach’s α = 0.92) loaded on ten items (10, 11, 12, 25, 28, 29, 30, 31, 37) that describe the affective tone of interactions and reciprocal attitudes of the parent and child, and that evaluate the presence of conflicts or dysfunctional patterns in their relationship, while considering the child’s development level. Healthy Relationship (Cronbach’s α = 0.83) loaded on four items (1R, 2R, 3R, 4R; “R” stands for “Reverse”), describing good aspects of the interaction between the parent and child. Contingent Problems (Cronbach’s α = 0.56) loaded on two items (6, 7), describing the possible impairment of a specific area of relational functioning and evaluating if the relation is flexible in different situations if a transient problem happens. Anxious Relationship (Cronbach’s α = 0.70) loaded on two items (32, 36), describing a relationship characterized by physical agitation and hyper-reactivity by both parent and child (Table 1).

Table 1 shows the correlations among factors for the four-factor model. Correlations were explored using Pearson’s coefficient of correlation. The correlations between Healthy Relationship and other factors were, as expected, always negative and, specifically, moderate for Dysfunctional Relationship (*r =* −0.55) and Anxious Relationship (*r =* −0.34) and low for Contingent Problems (*r =* −0.04). The correlation between Dysfunctional Relationship and Contingent Problems was low (*r =* 0.21), while the correlation between Dysfunctional Relationship and Anxious Relationship was moderate (*r =* 0.44). Lastly, the correlation between Contingent Problems and Anxious Relationship was low (*r =* 0.18). The four-factor model showed acceptable goodness of fit (GFI = 0.997; CFI = 1.000; TLI = 1.011; RMSEA = 0.000; SRMR = 0.029).

Regarding the Parent items, the eigenvalue-greater-than-one indicated two factors, the scree-plot showed two drops in eigenvalue size after the first and the second eigenvalue, and the PA criterion suggested one factor be retained. This information was interpreted as suggestive of three oblique factors, accounting for 64.16% of the variance in ratings, which was subsequently interpreted. Psychologically Unfit Parent (Cronbach’s α = 0.87) loaded on eight items (5R, 9, 17, 18, 23, 24, 27, 33; “R” stands for “Reverse”), evaluating how the parent is or is not able to manage the child, is sensitive to his or her signals, and is involved in the relationship. Intrusive Parent (Cronbach’s α = 0.67) loaded on two items (15, 34), evaluating how the parent interferes with the child’s own desires, and how much the parent is overprotective. Detached Parent (Cronbach’s α = 0.76) loaded on three factors (26, 38, 45), describing not only an insensitive parent but also a parent who ignores and refuses the child’s care and misunderstands the child’s need signals (Table 2). 

Table 2 shows the correlations among factors for the three-factor model. The correlations of Psychologically Unfit Parent with Intrusive Parent and Detached Parent were both moderate (*r* = 0.38 and *r* = 0.69, respectively). However, the correlations between Intrusive Parent and Detached Parent were low (*r* = 0.22). The three-factor model showed acceptable goodness of fit (GFI = 0.997; CFI = 1.000; TLI = 1.011; RMSEA = 0.000; SRMR = 0.030). 

Regarding the Child items, the eigenvalue-greater-than-one indicated two factors, the scree-plot did not show any drops in eigenvalue size, and the PA criterion suggested one factor to be retained. This information was interpreted as suggestive of two oblique factors accounting for 63.46% of the variance in ratings, which were subsequently interpreted. Withdrawal Child (Cronbach’s α = 0.79) loaded on four items (13, 19, 20, 42), evaluating if the child shows interactional impairments, due to disabilities or restricted affective expressions, or if he or she uses aggressive behavior as the only interactive modality. Anxious Child (Cronbach’s α = 0.53) loaded on two items (22, 35), describing how the child has difficulties in separation from the parent, and his or her tendency to be extremely compliant (Table 3).

Table 3 shows the correlations among the two-factor model. The correlation was low (*r* = 0.24). This last result confirms our choice of a two-factor model. The two-factor model showed acceptable goodness of fit (GFI = 0.995; CFI = 0.998; TLI = 0.993; RMSEA = 0.000; SRMR = 0.034).

### 3.2. Group Differences

The discriminating power of the P-CRS was analyzed using the six diagnostic categories of the clinical sample.

For this purpose, an ANOVA was conducted. Results are reported in Table 4. 

Regarding Interaction dimensions, the mean scores of Dysfunctional Relationship were found to be significantly different among the six clinical groups (ANOVA: *F* = 14.61, *p* < 0.001). Specifically, autism spectrum disorder and affective and relational disorders showed more Dysfunctional Relationship than others. The mean scores of Healthy Relationship significantly differed among the six diagnostic groups (ANOVA: *F* = 14.40, *p* < 0.001). Neurological disease, organic disease, genetic illness, and developmental delay (psychomotor, linguistic, cognitive delay) showed higher levels of Healthy Relationship functioning. The variance of Contingent Problems was not significantly different among the clinical groups (ANOVA: *F* = 1.40, *p* > 0.10); this dimension does not discriminate among different diagnoses. Finally, the mean scores of Anxious Relationship are significantly different among the six clinical groups (ANOVA: *F* = 9.40, *p* < 0.001). Affective and relational disorders were shown to be the disorders with the highest levels of anxiety in the parent-child relationship. 

Regarding the Parent dimensions, mean scores for Psychologically Unfit were found to be significantly different among the six clinical categories (ANOVA: *F* = 16.13, *p* < 0.001). We found the highest levels of this dimension in autism spectrum disorder and affective and relational disorders. The mean scores of Intrusive Parent were significantly different among the clinical groups (ANOVA: *F* = 4.38, *p* = 0.001); however, the difference was particularly significant between affective and relational disorders and developmental delay (psychomotor, linguistic, cognitive delay). Finally, mean scores for Detached Parent were also different among the six groups (ANOVA: *F* = 11.57, *p* < 0.001). Specifically, they were higher in affective and relational disorders than other groups (although not higher than autism spectrum disorder), and lower in developmental delay (psychomotor, linguistic, cognitive delay) and prematurity. 

Finally, regarding the Child dimensions, mean scores of Withdrawal Child were found to differ among the clinical groups (ANOVA: *F* = 25.56, *p* < 0.001). As expected, this dimension was highest in autism spectrum disorder and lowest in prematurity. The mean scores for Anxious Child were also different among the groups (ANOVA: *F* = 5.24, *p* < 0.001). They were lowest in neurological disease, organic disease, and genetic illness, and highest in affective and relational disorders.

## 4. Discussion

The P-CRS was set up to assess the quality of the parent-child relationship, from a clinician’s viewpoint, in a clinical setting. This study described the development and preliminary psychometric properties of the P-CRS, confirming the satisfactory reliability of the measure. After a first screening, in which items that did not respect the normality assumption were excluded, multiple parent-child relationship dimensions were validated through factor analysis, which provided coherent outcomes. 

Factor analysis identified different constructs within each macro-area. For “Interaction,” four dimensions emerged: Dysfunctional Relationship, Healthy Relationship, Contingent Problems, and Anxious Relationship. The scale, therefore, appears to be able to not only indicate how healthy or dysfunctional aspects are presented in the parent-child relationship, but also capture whether the interactions are characterized by a particularly anxious climate, which the literature has reported to be a potentially impairing element in child development [38]. Moreover, through the Contingent Problems dimension, the scale can evaluate whether such interactions show aspects of flexibility and adaptation, which refer to the concept of “interactive repair” that Tronick [39,40] considers to be a fundamental element in the development of intersubjective experience.

The factors were in line with dimensions identified by other measures also focused on the parent-child relationship. Dysfunctional Relationship and Healthy Relationship were in line with Adapted Relationship and Disordered Relationship of the PIR-GAS [4]. Anxious Relationship was in line with the Anxious/Tense factor of the RPCL [4]. However, unlike these scales, which were created to compare every relationship, using a given prototype to which a score of suitability is assigned, the P-CRS could provide a different contribution, as its purpose is to identify how several aspects that delineate a parent-child relationship intersect and combine for that specific relationship, reflecting an idiographic approach to assessment. 

Contingent Problems refer to Anders’s Classification of Mental Disorders [41]. Anders distinguished between relational perturbation, i.e., transient and short relational difficulties (e.g., feeding problems subsequent to weaning), relational disturbance that emerged only in a development area and is characterized by an inappropriate or insensitive interactional model (e.g., sleeping disorders, feeding disorders, etc.), and relational disorder that is revealed in a rigid, insensitive interaction model, which relates to several developmental areas and lasts more than three months. Contingent Problems can help clinicians understand the categories within which relational difficulties could be included. 

For “Parent”, three dimensions emerged: Psychologically Unfit Parent, Intrusive Parent, and Detached Parent. These dimensions are in line with those found by Ghanbari, Khodapanahi, Mazaheri, and Lavasani [42], who designed a scale to assess the quality of maternal caregiving through three main factors: “Confusion and Conflict,” “Sensitivity and Responsiveness,” and “Availability.” While we can consider “Sensitivity and Responsiveness” and “Availability” as the other faces of intrusiveness and detachment, the dimension of “Confusion and Conflict” could be considered similar to Psychologically Unfit Parent on the P-CRS. 

For “Child”, two different dimensions emerged: Withdrawal Child and Anxious Child. These aspects could be viewed as two extreme poles of a continuum that describes a child’s level of involvement in his or her relationship with a caregiver. 

In our opinion, the “Parent” and “Child” areas are innovative aspects of the P-CRS, as they allow clinicians to focus on the specific contributions of individual members in a dyad. 

These constructs not only emerged through factor analysis, but also provided clinical evidence that they are able to distinguish the caregiver and child’s different contributions to the relationship in six different diagnostic categories. The ANOVA showed that the first factor coherently distinguished dysfunctional aspects of the relationship among various kind of mental health disorders. Particularly, as expected based on the previous scientific literature, the results showed more relationship dysfunction for autism spectrum disorder [43]. Additionally, as expected, relationship dysfunction was found more for affective and relational disorders, rather than for neurological disease, organic disease, genetic illness, developmental delay (psychomotor, linguistic, cognitive delay), or prematurity. This result is in line with Maestro and colleagues [2], who observed that in affective disorders, the environment seems to have a crucial role and an impact on parent-child relationships. The second factor, Healthy Relationship, was found to be higher for neurological disease, organic disease, genetic illness, developmental delay, and prematurity, than for autism spectrum disorder or affective and relational disorders. This result could indicate that neurological disease, organic disease, genetic illness, developmental delay, and prematurity, even if damaging emotional development [44], cognitive ability [45], and psychomotor and linguistic development [46], do not necessarily impair parent-child relationships in the same way as autism spectrum disorder and affective and relational disorders. We could explain this result by assuming that only one difficulty in a specific developmental area is understandably more “manageable” for the parent, than an overt disorder that deeply affects the child’s overall interactional abilities. This result also indicates that the P-CRS could highlight the crucial role of the relational component in autism spectrum disorder, when compared with other neurological disorders. 

Regarding the “Parent” area, the finding that more “Psychologically Unfit” caregivers were found for children with autism spectrum disorder is in line with studies that have demonstrated that the parents of children with autism have more parenting difficulties [47]. Moreover, caregivers were found to be equally “Intrusive” in almost all clinical groups. In our opinion, this result could be interpreted as indicating that intrusive parental interactions are not necessarily linked with any particular type of child psychopathology. However, it is important to consider this aspect in relational assessments, due to the effect this interactional style can have on a child’s development and attachment model [48]. 

Furthermore, findings indicated that parents were particularly detached for children with affective and relational disorders, and less detached for children with developmental delay or prematurity. Parent detachment, in the form of neglect or insensitivity, may significantly contribute to affective and relational disorders. These overall results could be interpreted as indicating that when psychopathology is more related to neurological or physical problems than psychological problems, parenting is less affected, and parents seem to be more competent in the parent-child relationship. Moreover, research has observed that, for prematurity, parents are more involved and therefore less detached [49]. 

Regarding the “Child” area, the dimension of withdrawal captured one of the specific aspects of autism spectrum disorder that is shown in “deficits in the ability to initiate and to sustain reciprocal social interaction and social communication, and by a range of restricted, repetitive, and inflexible patterns of behavior and interests” [50], differentiating it from other types of psychopathology. These results are also in line with other studies [2,6] that have reported the specific low involvement of children with autistic traits or multisystem developmental disorder, and that have underlined the importance of this finding in supporting the need for specific parental programs to adapt their caregiving for these kind of children [2]. Otherwise, the anxious dimension was higher for affective and relational disorder than for other diagnostic categories. This result appeared to be in line with high levels of comorbidity between internalizing symptoms and feeding disorders [51] or sleeping disorders [52].

## 5. Conclusions

In conclusion, the P-CRS seems to be able to distinguish clinical conditions also in terms of the profiles of parents, children, and parent-child relationships.

This study’s outcomes were based on a sample of children with a psychological diagnosis and their parents. These findings have important clinical and research implications. In particular, the P-CRS is a useful and easy-to-use clinician-report, which could facilitate assessment and be useful to define possible outcomes. The results obtained from factor analysis need additional confirmation with a father-child sample, as well as a non-clinical sample, to be further validated. It could be interesting to see whether the P-CRS dimensions identified for mothers would be the same for fathers, considering that, as previously noted, some authors observed that mother-child and father-child interactions could be very different [21]. Moreover, it could be interesting to evaluate the diagnostic power of the measure, comparing clinical and non-clinical participants, to clarify if P-CRS factors could be useful in identifying different types of psychopathology. Additionally, it could be necessary to determine whether dimensions found in a clinical sample would be the same in a non-clinical sample, to ensure that the rating scale is able to account for aspects of the parent-child relationship outside a clinical setting. Moreover, it could be important to increase the sample size of clinical groups, to contribute to a clearer understanding of the roles of each dimension in the assessment of the disorder

Another limitation concerns the absence of analysis of the convergence with an independent measure of childhood behavior, which could better verify the construct validity of the P-CRS. 

Nevertheless, in light of what was observed through the ANOVA, the P-CRS can easily and quickly capture aspects of the parent-child relationship, as well as differentiate patterns that are typically present in some clinical situations. Moreover, the P-CRS can also be used for research purposes, as it enables the differentiation of individual contributions within the parent-child relationship. 

## Figures and Tables

**Table 1:** “Interaction” Area Exploratory Factor Analysis and Correlation Matrix Between Factors.

Items	F1	F2	F3	F4
30. The affective tone of the relationship is flat and constricted and characterized by withdrawal and sadness	**0.880**			
28. Interactions lack vitality and mutual pleasure	**0.744**			
29. The child and the parent appear detached, with little eye contact and little physical closeness	**0.738**			
12. In the report, there are dysfunctional patterns that appear deeply rooted	**0.645**			
25. There is a lack of coherence between the attitudes expressed by the parent toward the child and the observable quality of the interactions (predictability and/or reciprocity are absent in the sequence and order of exchanges)	**0.571**			
31. Interactions are tense and do not give a sense of tranquility, fun, or mutuality	**0.569**			
11. The relationship, even in the absence of conflict, may be inappropriate from the point of view of the child’s development (e.g., the child is treated as younger than his age)	**0.558**			
10. Most interactions between the child and the parent are conflicting and associated with a state of anxiety	**0.475**			
37. The report is characterized by rough and abrupt interactions, often devoid of emotional reciprocity	**0.392**			
8. If the parent and child are in conflict, this affects more areas of functioning	0.331			
1R. The interactions are pleasant for the child and for the parent and without reasons for anxiety		**−0.805**		
2R. The relationship is a stimulus for the growth of both the child and the parent		**−0.714**		
3R. The interactions are reciprocal and synchronous		**−0.660**		
4R. Sometimes the parent and the child may be in conflict, but this does not last for more than a few days		**−0.558**		
6. There is a disturbance in the relationship, but it is limited to only one aspect of functioning (e.g., power supply, play, regulation)			**0.661**	
7. If the child and the parent experience anxiety this lasts for a month or more; however, the relationship maintains an adaptive flexibility (e.g., through negotiation)			**0.609**	
32. The parent and the child present an anxious mood observable through motor tension, apprehension, agitation, facial expression, vocalization, or language				**0.719**
36. Both the parent and the child are hyper-responsive to one another				**0.627**
Cronbach’s α	0.92	0.83	0.56	0.70
Explained variance (%)	46.64	8.70	7.10	6.98
Correlations between factors (Pearson’s *r*)				
**F1**	1.000			
**F2**	−0.554	1.000		
**F3**	0.206	−0.036	1.000	
**F4**	0.441	−0.343	0.176	1.000

Bold values in the factorial matrix are those entered in the final factors (>350). F1 indicates “Dysfunctional Relationship”; F2 “Healthy Relationship”; F3 “Contingent Problems; F4 “Anxious Relationship”.

**Table 2:** “Parent” Area Exploratory Factor Analysis and Correlation Matrix between factors.

Items	F1	F2	F3
9. The parent is unable to sustain entire areas of the child’s functioning	**0.808**		
18. The parent makes requests that are not appropriate to the child’s level of development	**0.736**		
5R. The parent is able to fully support the functional capabilities appropriate to the age of the child	**0.703**		
24. The parent is insensitive and/or unresponsive to the child’s signals	**0.648**		
27. The parent is unable to adequately reflect the affective state of the child	**0.591**		
23. The parent shows sporadic or infrequent involvement or bonding	**0.566**		
33. The parent physically manipulates the child in a clumsy way	**0.490**		
17. The parent dominates the child, who reacts with provocative behavior	**0.488**		
34. The parent appears to be overprotective and frequently expresses concern for the child’s well-being, behavior, or development		**0.662**	
15. The parent often interferes with the child’s goals and wishes because they do not perceive the child as a separate individual with their own needs		**0.606**	
38. Especially when they see the child as too dependent and demanding, the parent is insensitive to the child’s signals			**0.810**
45. The parent misinterprets the baby’s crying as a deliberate negative reaction to them			**0.493**
26. The parent ignores, refuses, or is unable to comfort the child			**0.471**
Cronbach’s α	0.86	0.67	0.76
Explained variance (%)	47.117	10.372	6.670
Correlations between factors (Pearson’s *r*)			
**F1**	1.000		
**F2**	0.375	1.000	
**F3**	0.693	0.216	1.000

Bold values in the factorial matrix are those entered in the final factors (>350). F1 indicates “Psychologically Unfit Parent”; F2 “Intrusive Parent”; F3 ”Detached Parent”.

**Table 3:** “Child” Area Exploratory Factor Analysis and Correlation Matrix between factors.

Items	F1	F2
20. The child shows a narrow range of affective expressions	**0.935**	
13. The child has a disability that alters the parent’s ability to maintain an adequate relationship	**0.713**	
19. In the interaction with the parent, the child may appear to be late in motor skills and/or expressive language	**0.621**	
42. The child manifests provocative and aggressive behaviors toward the parent	**0.415**	
22. The child shows difficulty in separation		**0.807**
35. The child is condescending or anxious toward the parent in an unusual way		**0.523**
Cronbach’s α	0.76	0.53
Explained variance (%)	40.80	22.66
Correlations between factors (Pearson’s *r*)		
**F1**	1.000	
**F2**	0.241	1.000

Bold values in the factorial matrix are those entered in the final factors (>350). F1 indicated “Withdrawal child”; F2 “Anxious child”.

**Table 4:** Analysis of variance of each Parent-Child Relationship Scale (P-CRS) factor between six clinical conditions.

P-CRS Factors	Clinical Conditions											
	NOG (N = 36)		ASD (N = 45)		DD (N = 96)		PRE (N = 40)		ARD (N = 23)		OC (N = 28)	
	M	SD	M	SD	M	SD	M	SD	M	SD	M	SD
**Dysfunctional Relationship**	1.37 ^A^	0.69	2.24 ^B^	1.00	1.44 ^A^	0.61	1.28 ^A^	0.43	2.29 ^B^	0.78	1.63 ^A^	0.84
**Healthy Relationship**	3.69 ^A,C^	0.88	2.71 ^B^	0.98	3.73 ^A,C^	0.93	3.88 ^C^	0.70	2.61 ^B^	0.70	3.20 ^A,B^	1.16
**Contingent Problems**	2.06 ^A^	1.11	1.98 ^A^	1.06	2.01 ^A^	1.16	1.66 ^A^	1.03	2.17 ^A^	0.72	2.32 ^A^	1.17
**Anxious Relationship**	1.60 ^A^	0.72	1.98 ^A^	1.01	1.58 ^A^	0.84	1.93 ^A^	0.88	2.96 ^B^	0.96	2.04 ^A^	1.22
**Psychologically Unfit**	1.50 ^A^	0.63	2.22 ^B^	0.92	1.56 ^A^	0.55	1.33 ^A^	0.24	2.47 ^B^	0.83	1.73 ^A^	0.76
**Intrusive Parent**	2.07 ^A,B^	1.01	2.62 ^A,B^	1.11	1.97 ^A^	1.01	2.23 ^A,B^	0.80	2.85 ^B^	1.10	2.18 ^A,B^	1.22
**Detached Parent**	1.25 ^A,B^	0.55	1.64 ^B,C^	0.71	1.15 ^A^	0.40	1.18 ^A^	0.35	1.99 ^C^	0.93	1.55 ^B^	0.79
**Withdrawal Child**	2.11 ^A,B^	1.12	3.65 ^C^	1.24	2.14 ^A,B^	1.08	1.48 ^A^	0.68	2.61 ^B^	1.23	1.41 ^A^	0.56
**Anxious Child**	1.32 ^A^	0.47	1.72 ^A,B^	0.84	1.66 ^A,B^	0.81	1.70 ^A,B^	0.84	2.39 ^C^	1.12	2.00 ^B,C^	1.10

Values marked with different letters (A, B, C) are significantly different from each other (*p* < 0.003). Values marked with the same letters indicate no differences between groups. Pairwise comparisons were significant at a Bonferroni corrected alpha level of 0.003 (derived by dividing 0.05 by 15); NOG = Neurological disease, organic disease, genetic illness; ASD = Autism Spectrum Disorder; DD = Developmental delay (psychomotor, linguistic, cognitive delay); PRE = Prematurity; ARD = Affective and Relational Disorders; OC = Other Conditions.

## Supplementary Materials

The following are available online at https://www.mdpi.com/1660-4601/17/10/3458/s1, Table S1: Descriptive Statistical Analysis of P-CRS items.
