# Supplementary material for: P-CRS: A Clinical Scale to Assess the Parent-Child Relationship in Infancy and Early Childhood
Source: Int J Environ Res Public Health. 2020 May 15;17(10):3458. doi: 10.3390/ijerph17103458 (PMC7277328; doi:10.3390/ijerph17103458)
Supplement: Supplementary file 1 [file ijerph-17-03458-s001.pdf]

Supplementary

Table S1. Descriptive Statistical Analysis of P-CRS items.

		M	SD	Skewness	Kurtosis
1.	The interactions are pleasant for the child and for the parent and without reasons for anxiety [a]	2.54	1.18	0.21	−0.91
2.	The relationship clearly promotes the growth of both child and parent [a]	2.43	1.19	0.45	−0.68
3.	Interactions are frequently reciprocal and synchronous [a]	2.71	1.21	0.07	−1.01
4.	Sometimes the parent and the child may be in conflict, but this does not last for more than a few days [a]	2.63	1.40	0.41	−1.08
5.	The parent is able to fully support the functional capabilities appropriate to the age of the child [b]	2.54	1.22	0.46	−0.68
6.	There is a disturbance in the relationship, but it is limited to only one aspect of functioning (e.g., power supply, play, regulation, etc.) [a]	2.13	1.41	0.94	−0.50
7.	If the child and the parent experience anxiety this lasts for a month or more; however, the relationship maintains an adaptive flexibility (e.g., through negotiation) [a]	1.88	1.20	1.18	0.28
8.	If the parent and child are in conflict, this affects more areas of functioning [a]	2.17	1.30	0.76	−0.62
9.	The parent is unable to sustain entire areas of the child's functioning [b]	1.97	1.28	1.16	0.13
10.	Most interactions between the child and the parent are conflicting and associated with a state of anxiety [a]	1.69	1.10	1.58	1.55
11.	The relationship, even in the absence of conflict, may be inappropriate from the point of view of the child's development (e.g., the child is treated as younger than his age) [a]	2.09	1.36	0.89	−0.59
12.	In the report, there are dysfunctional patterns that appear deeply rooted [a]	1.88	1.28	1.26	0.31
13.	The child has a disability that alters the parent's ability to maintain an adequate relationship [c]	2.21	1.55	0.87	−0.86
14.	Interactions are so frequently disturbed that the child is in imminent danger of physical harm [a*]	1.18	0.59	3.76	14.87
15.	The parent often interferes with the child's goals and wishes because they do not perceive the child as a separate individual with their own needs [b]	1.92	1.10	0.92	−0.23
16.	The parent dominates the child and he/she may appear submissive with compliant behaviors [b*]	1.24	0.66	3.45	13.62

17. The parent dominates the child, who reacts with provocative behavior [b]	1.42	0.85	2.26	4.94
18. The parent makes requests that are not appropriate to the child's level of development [b]	1.80	1.15	1.31	0.72
19. In the interaction with the parent, the child may appear to be late in motor skills and / or expressive language [c]	2.43	1.51	0.52	−1.23
20. The child shows a narrow range of affective expressions [c]	2.12	1.41	0.95	−0.49
21. The parent may perceive the child as a partner or as a peer [b*]	1.24	0.72	3.36	10.88
22. The child shows difficulty in separation [c]	2.14	1.31	0.81	−0.56
23. The parent shows sporadic or infrequent involvement or bonding [b]	1.28	0.70	2.78	7.61
24. The parent is insensitive and / or unresponsive to the child's signals [b]	1.28	0.66	2.62	7.03
25. There is a lack of coherence between the attitudes expressed by the parent toward the child and the observable quality of the interactions (predictability and / or reciprocity are absent in the sequence and order of exchanges) [a]	1.69	1.09	1.57	1.63
26. The parent ignores, refuses, or is unable to comfort the child [b]	1.40	0.80	2.28	5.25
27. The parent is unable to adequately reflect the affective state of the child [b]	1.94	1.12	0.94	−0.13
28. Interactions lack vitality and mutual pleasure [a]	1.60	0.95	1.64	2.08
29. The child and the parent appear detached, with little eye contact and little physical closeness [a]	1.47	0.86	1.91	3.06
30. The affective tone of the relationship is flat and constricted and characterized by withdrawal and sadness [a]	1.37	0.82	2.66	7.34
31. Interactions are tense and do not give a sense of tranquility, fun, or mutuality [a]	1.67	1.05	1.55	1.59
32. The parent and the child present an anxious mood observable through motor tension, apprehension, agitation, facial expression, vocalization, or language [a]	2.00	1.21	0.98	−0.10
33. The parent’s physical handling of the child may be awkward [b]	1.25	0.64	2.96	9.29
34. The parent appears overprotective and expresses frequent concern regarding the child’s wellbeing, behavior, or development [b]	2.53	1.34	0.39	−1.03
35. The child is condescending or anxious toward the parent in an unusual way [c]	1.32	0.74	2.66	7.21
36. Both the parent and the child are hyper-responsive to one another [a]	1.73	1.02	1.22	0.54

37. The report is characterized by rough and abrupt interactions, often devoid of emotional reciprocity [a]	1.25	0.64	2.93	9.12
38. Especially when they see the child as too dependent and demanding, the parent is insensitive to the child's signals [b]	1.31	0.75	2.84	8.40
39. The parent taunt or tease the child [b*]	1.08	0.37	5.51	34.61
40. Interactions have a hostile or angry edge [a*]	1.23	0.65	3.17	10.35
41. The child may exhibit fearful, vigilant, and avoidant behaviors [c*]	1.12	0.46	4.81	27.79
42. The child manifests provocative and aggressive behaviors toward the parent [c]	1.57	1.04	1.81	2.38
43. In the relationship there are severe verbal and/or emotional abusive contents [a*]	1.04	0.29	7.72	62.77
44. The content of verbal/emotional abuse by the parent is intended to severely belittle, blame, attack, overcontrol, and/or reject the child [b*]	1.11	0.46	5.04	29.41
45. The parent misinterprets the baby's crying as a deliberate negative reaction to them [b]	1.38	0.77	2.09	3.54
46. The parent physically harms the child [b*]	1.08	0.39	5.42	31.58
47. The parent has difficulty limits to the child in a non-violent manner [b*]	1.26	0.75	3.46	12.66
48. The relationship involves a lack of compliance with physical boundaries and an extreme sexualized intrusiveness [a*]	1.03	0.23	9.92	115.57
49. The parent shows sexually seductive and overstimulating behavior [b*]	1.01	0.11	9.34	85.96
50. The parent neglects the child with respect to his physical and emotional needs [b*]	1.19	0.65	3.93	16.18

[a] Items marked with an "a" belong to "Interaction Area", [b] Items marked with a "b" belong to "Parent Area", [c] Items marked with a "c" belong to "Child Area", * Items marked with an asterisk have been delated because their distribution does not respect the normality assumption.
